# Competitive Effects Hinder the Recolonization of Native Species in Environments Densely Occupied by One Invasive Exotic Species

**DOI:** 10.3389/fpls.2018.01261

**Published:** 2018-09-04

**Authors:** Thaisa S. Michelan, Sidinei M. Thomaz, Fabielle M. Bando, Luis M. Bini

**Affiliations:** ^1^Departamento de Biologia, Universidade Estadual de Maringá, Nupelia, Maringá, Brazil; ^2^Laboratório de Ecologia e Conservação, Instituto de Ciências Biológicas, Universidade Federal do Pará, Belém, Brazil; ^3^Departamento de Ecologia, Instituto de Ciências Biológicas, Universidade Federal de Goiás, Goiânia, Brazil

**Keywords:** competition, non-native macrophytes, Poaceae, density-dependent effect, resource competition

## Abstract

The responses of native plants to competition with invasive plants depend mainly on the density of the invasive plants and on the ability of the native plants to compete for resources. In this study, we tested the influence of the invasive exotic *Urochloa arrecta* (Poaceae) on the early colonization of two native species (*Pontederia cordata* and *Leersia hexandra*) of aquatic macrophytes. Our hypotheses were (i) the competitive effects of *U. arrecta* on the native species *P. cordata* and *L. hexandra* are density-dependent and that (ii) these species respond differently to competitive interactions with the invasive species. We conducted the experiments in a greenhouse and in the field, in a tropical reservoir. The biomass of *U. arrecta* (ranging from 206.2 to 447.1 g) was manipulated in the greenhouse in trays with different densities. After the establishment of the invasive species, we added *P. cordata* and *L. hexandra* propagules to each tray. In the field, a propagule of *P. cordata* was planted in 36 sites with different densities of *U. arrecta*. The biomass and length of the natives and the biomass of the invasive species were measured in the greenhouse and in the field experiments. The biomass and length of the native plants decreased with increasing biomass of the exotic species in both experiments, showing that the competition between *U. arrecta* and native species depends on the density of the exotic species. The root:shoot ratio of *L. hexandra* decreased with increasing *U. arrecta* biomass, but the opposite occurred for *P. cordata*. These results indicate that native species exhibit different strategies of biomass allocation when interacting with *U. arrecta*. The strong competitive effects of *U. arrecta* and the different responses of the native species help to explain the reduced diversity of native macrophytes observed in sites colonized by *U. arrecta*. The results also suggest that in a scenario of dominance of exotic species, recolonization by native macrophytes is unlike to occur naturally and without human interventions that reduce the biomass of the exotic species.

## Introduction

In general, only a small fraction of introduced species become successfully established and exhibit population growth to the point of becoming “invasive" (Levine, 2008; Davis, 2009). When they do become invasive, they can reduce the richness and abundance of native species (Madsen et al., 1991; Daehler and Strong, 1994; Roberts et al., 1999; Michelan et al., 2010b). In addition, they can even change the environmental conditions of the invaded sites (Pyšek et al., 2008; Strayer, 2010), causing ecological and economic damage (Richardson and Pyšek, 2008; Carey et al., 2016; Cuassolo et al., 2016). Invasion success depends on multiple factors, including species-specific traits (e.g., growth rate, competitiveness and dispersal ability; Rejmánek, 2011) and the characteristics of the invaded ecosystem (e.g., environmental conditions, disturbances and diversity; Fridley, 2011).

The impacts of invasive species on native species depend largely on the abilities of interacting species to compete for resources (Seabloom et al., 2003; Blindow et al., 2016). Competition is an important biological interaction that influences the structure and development of plant communities (Kiaer et al., 2013; Blindow et al., 2016). Additionally, species respond differently to competition depending on the abiotic conditions and on the density of each population (Gopal and Goel, 1993; Nunes and Camargo, 2017). As a direct result of competition with invasive species, one can predict changes in the structure of invaded communities and a decrease in biodiversity at local and regional scales (Michelan et al., 2010b; Powell et al., 2011, 2013; Amorim et al., 2015).

Competition among plants occurs predominantly by nutrient (“root competition”) and/or light acquisition (“above-ground competition”). The roots and shoots of the plants acquire different resources from the environment, and some studies therefore try to separate the effects of the competition of each plant part (Wang et al., 2008; Kiaer et al., 2013; Richter and Gross, 2013). One of the methods to evaluate which organ is most involved in the competition is to use root:shoot biomass ratio (Robinson et al., 2010). High values of this ratio indicate that competition for nutrients and water (by root) is more important, while lower values indicate greater competition for light (Wang et al., 2008; Craine and Dybzinski, 2013; Kiaer et al., 2013; Richter and Gross, 2013; but see Cahill, 2003 for another point of view).

Coexistence between species under natural conditions can be facilitated by several mechanisms, such as disturbances and trade-offs between competitive and dispersal abilities (Grime, 1979; Connell, 1983). However, the competitive effects of invasive species occurring at high densities may be so intense that, at least at fine spatial scales, native species are excluded by competition (Madsen et al., 1991). Yet, little is known about the tolerable limits of the biomass of invasive macrophytes for the recolonization of native macrophyte species. Thus, it is important to evaluate the competitive interactions between native and invasive macrophyte species at different biomasses of the latter. Studies employing this approach would help to identify native species with higher potential for recolonizing environments dominated by invasive species and to identify thresholds of invasive biomass that allow native recolonization and survival.

Many species belonging to the family Poaceae are highly invasive in several aquatic ecosystems (Bunn et al., 1998; Bell et al., 2011; Mugwedi et al., 2015). In general, they have greater competitive effects than species of other groups, such as herbaceous and leguminous plants (Kiaer et al., 2013). This is also the case for *Urochloa arrecta* (Hack. ex T. Durand & Schinz) Morrone & Zuloaga, a species native to Africa, which has colonized tropical and subtropical aquatic ecosystems. In particular, this species is invading and causing ecological impacts in various Brazilian aquatic ecosystems (Pott et al., 2011; Fernandes et al., 2013; Amorim et al., 2015). *U. arrecta* forms large patches, accumulates large amounts of biomass in littoral zones (Michelan et al., 2010b; Fernandes et al., 2013; Amorim et al., 2015), regenerates rapidly after disturbances (Michelan et al., 2010a) and can thrive even in relatively oligotrophic environments with nutrient-poor sandy substrates (Fasoli et al., 2015). However, there is a paucity of experimental studies evaluating the competitive effects of *U. arrecta* on individual macrophyte species.

In this study, we investigated the biomass-dependent effects of *U. arrecta* on the recolonization of two native species of macrophytes (*Pontederia cordata* L. and *Leersia hexandra* Sw.) and tested whether the competitive effects on them differ. First, we developed a greenhouse experiment to test the effects of *U. arrecta* on the biomass and on the root:shoot ratio of the two native species. Then, we repeated the experiment in the field, using *P. cordata* as a focal species, to test the generality of our results obtained in the greenhouse. We tested the hypotheses that (i) the competitive effects of *U. arrecta* on *P. cordata* and *L. hexandra* depend on the invasive biomass and (ii) that native species respond differently to these effects. These hypotheses were postulated because previous studies in the field showed that the native macrophyte biomass decreases in the presence of *U. arrecta* (Michelan et al., 2010b) and that the frequencies of co-occurrence between native macrophytes and *U. arrecta* are species-specific, indicating that native species may respond differently to increasing invasive biomass (Thomaz and Michelan, 2011). We predicted that the effects of *U. arrecta* on *L. hexandra* would be higher than those on *P. cordata* because of the morphological similarity and phylogenetic relationship between the first pair of species. Consequently, they should use resources more similarly, which intensifies competition and reduces the chances of co-existence (Chesson and Kuang, 2008; Rejmánek, 2011). Finally, to place our results in a broader context, we compared our results with those obtained in a recent meta-analysis (Jauni and Ramula, 2015).

We believe that the use of *U. arrecta* as a model plant in our study contributes to a broader view regarding the impacts of exotic plants on native species because this species belongs to the family Poaceae, which is responsible for the greatest ecological impacts among invasive plants (Kiaer et al., 2013). In addition, by conducting experiments in a greenhouse and in the field, we believe that our outcomes can be useful to assess whether the former can be extrapolated to nature, an issue that has been questioned by some investigations that highlight the shortcomings of microcosm experiments (e.g., Wilson and Keddy, 1991).

## Materials and Methods

We performed two experiments employing an additive design (Gibson et al., 1999) to assess the ability of native species to colonize and grow in sites with different biomasses of *U. arrecta*, one experiment in a greenhouse at the State University of Maringá (Paraná State, Brazil) and the other *in situ* in the Rosana Reservoir (Paraná/São Paulo, Brazil; 22°39′26.19″ S 52°46′52.35″ W; see **Supplementary Figure S1** for photos of the experiment in a greenhouse and *in situ*).

### Greenhouse Experiment

*Urochloa arrecta* (exotic), *P. cordata*, and *L. hexandra* (natives) were used in our greenhouse experiment. The native species were selected based on different levels of co-occurrence with *U. arrecta* (details about this selection in Michelan et al., 2013). *L. hexandra* (Poaceae species often found to co-occur with *U. arrecta*) is a perennial species that can grow vigorously in aquatic ecosystems (Pott and Pott, 2000; Moreira et al., 2011). The Pontederiaceae *P. cordata* has low level of co-occurrence with *U. arrecta* and is a perennial herbaceous species that can also form dense stands (Pott and Pott, 2000). Both species reproduce sexually and asexually (by stems and rhizomes). *P. cordata* rhizomes can survive to fire or dry seasons (Pott and Pott, 2000). The macrophytes were collected in the Rosana Reservoir and taken to the greenhouse.

We used trays (0.30 m × 0.37 m × 0.14 × m) that were filled halfway with sediment and maintained with a 3–5 cm water layer. The water was replaced with tap water whenever necessary. To create a gradient of *U. arrecta* biomass, we added fragments with two nodes each of *U. arrecta* from the apical stems, at densities of 0, 5, 10, 15, 20, 25, and 30 fragments per tray, with five replicates for each treatment, amounting to 35 trays. Trays were randomized inside the greenhouse to offset any undetected environmental variation.

A clear gradient in biomass, a necessary condition to test our hypotheses, was formed by 200 days after planting *U. arrecta* (0–450 g DW m^−2^). Then, we added one propagule of *P. cordata* and one of *L. hexandra* at the opposite extremes of each tray (separated from each other by ca. 30 cm). These propagules of *P. cordata* and *L. hexandra* were collected in the Rosana Reservoir and brought to the greenhouse, where we removed their leaves and roots in order to allow all plants to start to grow at similar conditions. In addition, we selected propagules with similar weights. The distance between the native species in the microcosms (ca. 30 cm) was assumed to be enough to avoid interaction between them. Although we did not measure the survival rates of the propagules of the native species during the course of the experiment, all propagules survived in our experiment and even those planted in more dense microcosms formed small individuals.

The experiment was completed 3 months after the introduction of the native species. The length of *P. cordata* and the average length of shoots generated by *L. hexandra* were measured with a tape (cm). Afterward, the biomass of each species was washed to remove sediment and was separated into shoots and roots. After drying in an oven (70°C, until constant weight), the dry mass of roots and shoots of each species was measured on a scale with a precision of 0.01 g. We emphasize that the experimental design of this study simulates a situation in which the recolonization by native aquatic macrophyte species occurs after the occupation of an invasive exotic species that is dominant in an ecosystem, a common situation in several Neotropical aquatic environments.

### Field Experiment

The field experiment was conducted in one arm of the Rosana Reservoir (between 22°39′19.29″ S; 52°46′58.93″ W–22°40′27.19″ S; 52°47′10.32″ W and 22°39′32.42″ S; 52°46′36.52″ W–22°40′26.18″ S; 52°46′47.43″ W; Brazil), near the sites where the native macrophytes were collected for the greenhouse experiment. We first selected 50 sites (squares of 0.09 m^2^–0.3 m × 0.3 m) with different densities of *U. arrecta*. These sites were identified and demarcated with stakes, and a propagule of *P. cordata* was planted in each site. The *P. cordata* propagules were treated the same way as those used in the greenhouse (see details above for the greenhouse experiment). We monitored the field experiment at every week and observed that the propagules of *P. cordata* were consumed by herbivores in 14 sites. Thus, 3 months after the establishment of the native species, only 36 sites were used in this study.

At the end of the experiment (90 days), the individuals of *P. cordata* and the shoots of *U. arrecta* in an area of 0.09 m^2^ (0.3 m × 0.3 m) around the native species were collected. For each site, the species were separated and washed, packed and placed in an oven at 70°C until reaching a constant weight. The dry shoot biomass of *U. arrecta* and the shoot and root biomass of *P. cordata* were obtained by using a precision scale with an accuracy of 0.01 g.

### Data Analysis

Following Goldberg and Scheiner (2001), we used an analysis of covariance (ANCOVA) for each response variable (i.e., total biomass, length and root:shoot ratio of native plants). In each ANCOVA model, the quantitative and categorical predictor variables were the shoot biomass of *U. arrecta* and the native species identity (*L. hexandra* and *P. cordata*), respectively. The response variables were standardized to the values of these variables expected in the absence of *U. arrecta*. Thus, we expressed them as log response ratios, *lnRR* = ln(*Y*_u_/*Y*_c_), where *Y*_c_ was the value of a response variable without *U. arrecta* and *Y*_u_ was the value of this response variable under the influence of *U. arrecta*. To estimate *Y*_c_, we used the mean values of the response variables in the control experimental units. Thus, *lnRR* is equal to 0.0 in the absence of competitive effects, and the more negative *lnRR* is, the higher the effect of *U. arrecta* in limiting the growth of the natives. As our objective was to analyze the competition between the invasive and native species and to evaluate whether they responded differently to the competition effect in a scenario of exotic dominance, we chose to remove the sites with the absence of the invasive species (but see the **Supplementary Figure S2** for the results based on the entire gradient of *U. arrecta* biomass—with the addition of treatment 0—absence of competition).

In the field experiment, the effect of *U. arrecta* biomass on *P. cordata* biomass was tested using a second-order polynomial regression due to the non-linearity of the data. All statistical analyses were performed in R (R Development Core Team, 2014).

### Comparison of the Results With Those Obtained in the Literature

To contrast our results with those obtained in the literature, we first transformed the Pearson correlation coefficient (*r*) between the total biomass of the invasive species and the total biomass of the native species into Cohen’s *d* using: *d* =2r/$\sqrt{1-r^{2}}$ (Borenstein et al., 2009). The variance of *d* (*V_d_*) is given by: *V_d_* = 4*V_r_*/(1 − *r*^2^)^3^, where *V_r_* is the variance of *r* (*V_r_* = (1 − *r*^2^/*n* − 1) and *n* is the sample size. Finally, *d* was transformed into Hedges’ *g* after multiplying *d* by a correction factor *j* (see Eq. 4.22 in Borenstein et al., 2009). The variance of *g* was estimated by *V_g_* = *j*^2^ ×*V_d_*. Second, we compared the values of *g* estimated in our study with the results of a recent meta-analysis conducted by Jauni and Ramula (2015). This meta-analysis was based on 75 competition studies between exotic and native species. These studies (observational and experimental) evaluated “how exotic plant species influence the fitness components of native plants” (Jauni and Ramula, 2015) and were based on the following response variables: establishment (i.e., germination), growth rate, biomass, reproductive success and survival (or mortality). Fifty-eight exotic species were included, and it is important to note that no study with *U. arrecta* was used in this meta-analysis, ensuring independent results. We focused our comparison on results obtained for biomass, based on studies with 19 exotic species (mainly from the order Poales) and 36 native species (see Figure 1 of Jauni and Ramula, 2015). In general, the approach of quantitatively comparing the results obtained in an experiment with those obtained in a meta-analysis can be considered a type of cumulative meta-analysis (Leimu and Koricheva, 2004).

## Results

### Greenhouse Experiment

**FIGURE 1:**
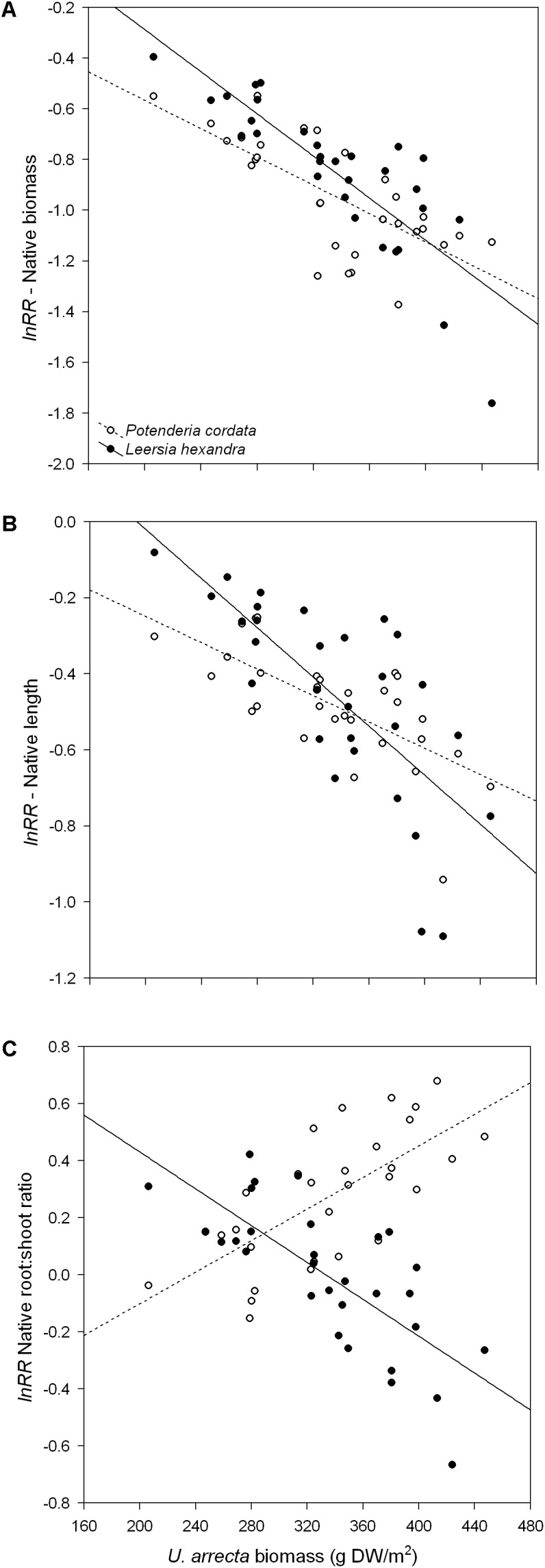
Relationship between the shoot biomass of *U. arrecta* and different traits of two species of native macrophytes. Shown are the results for **(A)** total biomass, **(B)** length, and **(C)** root:shoot ratio.

We found that the total biomass of both native species significantly decreased with the increase in the biomass of *U. arrecta*. The slopes of the relationships did not differ significantly (test of parallelism: *F*_1,56_ = 3.47; *P* = 0.0676; **Figure 1A**). However, the coefficient of determination of the model for *L. hexandra* (*R*^2^ = 0.70; *P* < 0.001) was substantially higher than that observed for *P. cordata* (*R*^2^ = 0.51; *P* < 0.001).

Similar to what we found for biomass, the native species’ length decreased significantly with the increase in the biomass of *U. arrecta* (**Figure 1B**). However, the slope of the relationship between the biomass of *U. arrecta* and length of *L. hexandra* (*b* = −0.0032 ± 0.0006 SE; *R*^2^ = 0.54; *P* < 0.001) was significantly steeper (test of parallelism: *F*_1,56_ = 5.14; *P* = 0.0272) than that for *P. cordata* (*b* = −0.0017 ± 0.0003 SE; *R*^2^ = 0.48; *P* < 0.001).

The effects of the *U. arrecta* biomass on the root:shoot ratio clearly differed between the two native species (*F*_1,56_ = 60.58; *P* < 0.001; **Figure 1C**), being positive for *P. cordata* (*b* = 0.0028 ± 0.0005 SE; *R*^2^ = 0.49; *P* < 0.001) and negative for *L. hexandra* (*b* = −0.0032 ± 0.0006 SE; *R*^2^ = 0.54; *P* < 0.001). Despite the opposite effects, the magnitudes of the slopes were similar. These results indicate that *L. hexandra* invests more in shoots, while *P. cordata* invests more in belowground structures (roots), with an increasing degree of competition with *U. arrecta*.

### Field Experiment

**FIGURE 2:**
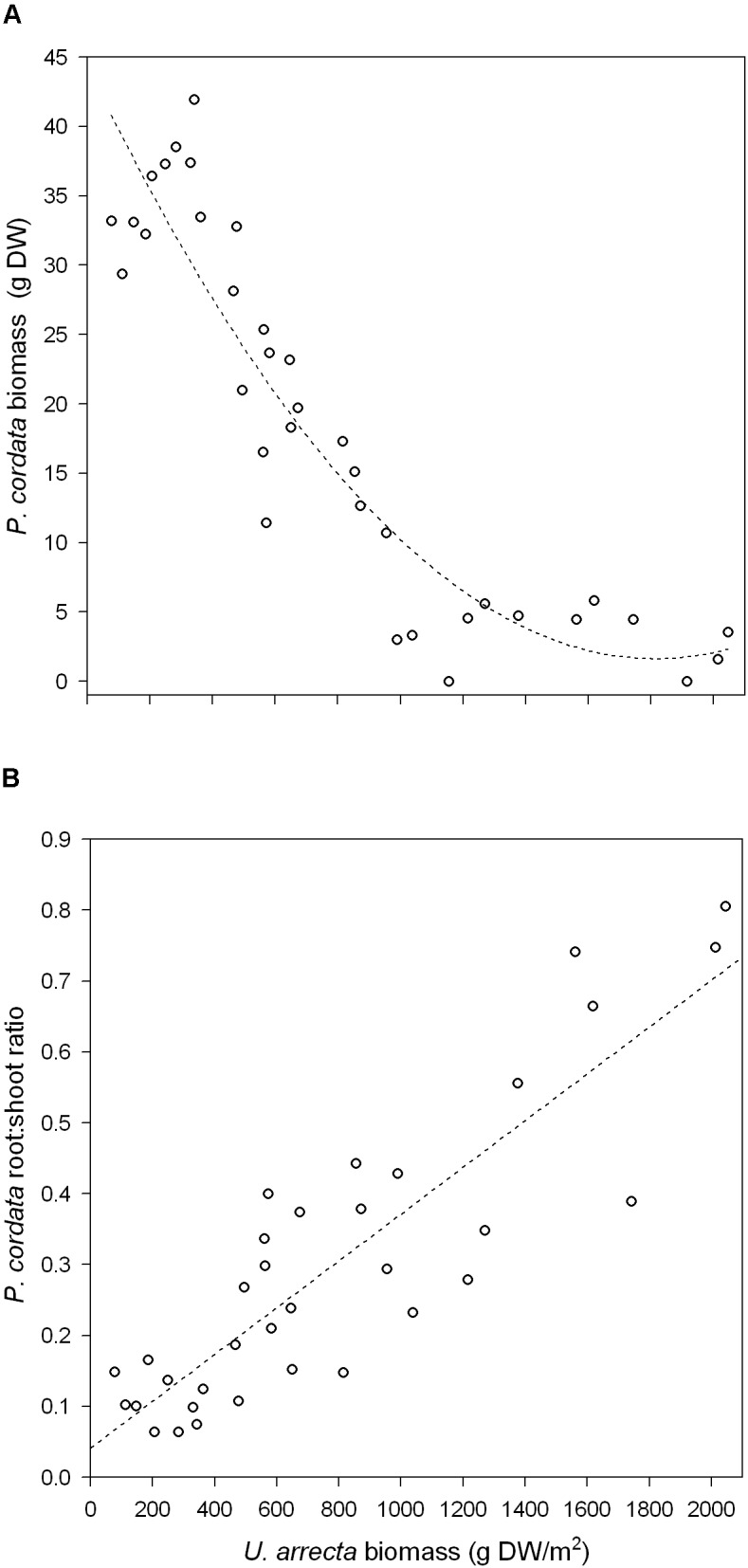
Relationship between the shoot biomass of *U. arrecta* and the biomass **(A)** and root:shoot ratio **(B)** of *Pontederia cordata* (data from the *in situ* experiment).

The results obtained in the field experiment with *P. cordata* followed the pattern found in the greenhouse. The biomass of *U. arrecta* negatively affected the biomass of *P. cordata* (*b* = −0.0472 ± 0.006 SE; *R*^2^ = 0.85; *P* < 0.001; **Figure 2A**). The root:shoot ratio of the biomass of this native species was positively and significantly affected by the biomass of the invasive species (*b* = 0.003 ± 0.0001 SE; *R*^2^ = 0.76; *P* < 0.001; **Figure 2B**).

### Comparison of the Results With Those Obtained in the Literature

**FIGURE 3:**
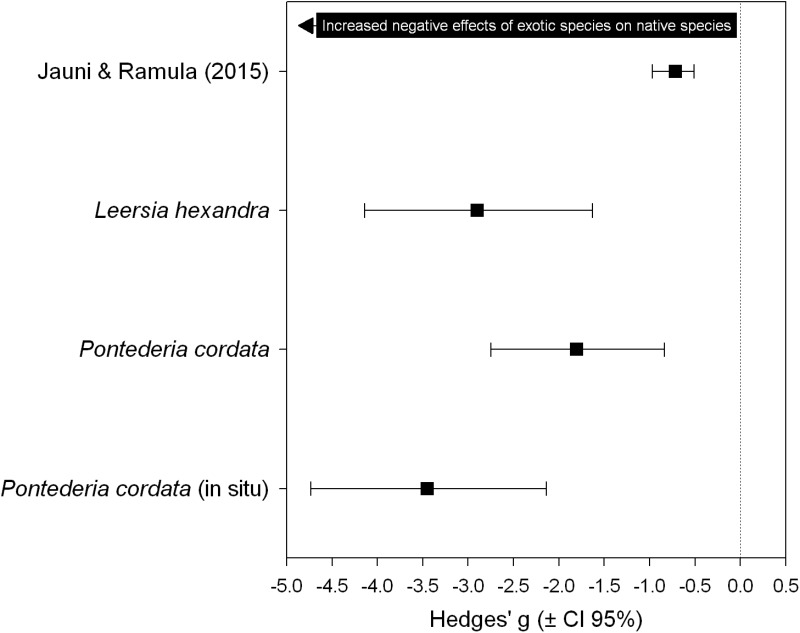
Cumulative effect size estimated by Jauni and Ramula (2015) and effect sizes estimated in this study. These assess the effects of invasive exotic plants on the biomass of native plants.

The effect of *U. arrecta* on native species was substantially larger than the effects reported in the meta-analysis of Jauni and Ramula (2015). The results of this comparison also indicate that the negative effect of *U. arrecta* on *L. hexandra* was greater than that estimated for *P. cordata* in the greenhouse experiment. However, the largest effect size was estimated for *P. cordata* when the experiment was carried out in the field, despite the high overlap between the confidence intervals (**Figure 3**).

## Discussion

We found that the competitive effects of *U. arrecta* on *P. cordata* and *L. hexandra* are biomass-dependent, corroborating our first hypothesis. Most importantly, in accordance with our second hypothesis, we showed that the native species responded differently to the competitive interaction with the invader, at least in terms of plant height and root:shoot ratios. In addition, it is likely that the increase in *U. arrecta* biomass increases the competition for nutrient acquisition more in *P. cordata* than in *L. hexandra*, given that the former species invested more in root growth when in greater competitive interaction with *U. arrecta* than the latter. However, although these native species responded differently to the competitive interaction with the invader, *L. hexandra* was not the most negatively affected by *U. arrecta* (as suggested by similar responses in terms of biomass production), which contradicts our prediction in this regard.

The negative effects of *U. arrecta* on native species were even stronger in the field experiment, where the growth of *P. cordata* was nearly suppressed at high densities of *U. arrecta* (see **Figure 2A**). These results, along with those obtained in the greenhouse, indicate that high densities of the invasive species decrease recolonization success by native species, which may explain the pattern of reduced macrophyte diversity with the increase of *U. arrecta* biomass at small spatial scales (Michelan et al., 2010b; Amorim et al., 2015). Our findings agree with studies carried out with other invasive species that showed the importance of density in the establishment success of native species, mainly in controlled experiments (Doyle et al., 2003; Martin and Coetzee, 2014). Thus, in field conditions, the effects of exotic species on the growth of native species are likely to be much stronger than the effects measured in greenhouse experiments. For example, the biomass that *U. arrecta* may attain in the field (approx. 7000 g DW m^−2^; Carniatto et al., 2013) is much higher than the highest biomass in our experiment (approx. 2000 g DW m^−2^).

Macrophytes respond to competition (Burns and Winn, 2006) and other environmental factors, such as increase in water level (Gomaa and AbdElgawad, 2012), by shoot elongation. The elongation (or etiolation) of terrestrial and aquatic plants is, in general, a response to light limitation (e.g., Goldsborough and Kemp, 1988; Paciullo et al., 2008, 2011; Li et al., 2011). However, our results indicate a reduction in the length of native species with increased competition (and shading) by *U. arrecta* (see **Figure 1B**). Thus, our results agree with other studies showing that not all species are able to etiolate in the face of light competition. For example, Burns and Winn (2006) demonstrated that competition reduced the lengths of two grass species. A plausible explanation for the lack of etiolation in face of competition, applicable only to *L. hexandra*, is that the light limitation was offset by the increased investment in the biomass of the shoots, as shown by the results for the root:shoot ratio (see below). By contrast, *P. cordata* has broader leaves than *L. hexandra*, and thus, increased light acquisition may be obtained by increases in leaf area instead of etiolation. These factors may also explain the steeper reduction in length for *L. hexandra* than for *P. cordata* along the invasive biomass gradient.

The native species responded differently to the increase in *U. arrecta* biomass in terms of investment in belowground or aboveground structures (see **Figure 1C**), revealing different strategies to overcome competition with the invasive species. The increase in plant density exacerbates competition, which may occur for space, nutrients and/or light (Witkowski, 1991; Daehler, 2003; Doyle et al., 2003; Davis, 2009). Increased investment in shoots indicates a predominance of competition between aboveground plant structures, while high investment in roots indicates dominance of competition between belowground structures (e.g., Berendse and Möller, 2009; Janeček et al., 2014). Based on this premise, our results indicate that *L. hexandra* growth becomes increasingly limited by light availability over a gradient of *U. arrecta* biomass, while *P. cordata* growth becomes increasingly limited by nutrients and space over the same biomass gradient. The largest investment in shoots by *L. hexandra* at high densities of the invasive probably occurs because Poaceae are, in general, highly sensitive to shading conditions. Allocation to shoot biomass, relative to root biomass, is likely to be a response to light limitation under high competition, as observed for other herbaceous species (Gibson et al., 2004; Awan et al., 2015). On the other hand, increased allocation to roots in *P. cordata* indicates a response to root competition (for other examples, see Bakker and Wilson, 2001; Schiffers et al., 2011; Zhu et al., 2015).

Despite the changes in biomass allocation of the native species over the competition gradient, there was a reduction of approximately 90% of the native species biomass at high biomass of *U. arrecta*, and the reduction was even higher for *P. cordata* in the field. The significant reduction in the growth of native species when exotic species are dominant, as simulated in our experiments, supports the model of preemptive competition, as found in other studies (Grace, 1987; Seabloom and van der Valk, 2003; Moore and Franklin, 2012; Moore et al., 2014). In practical terms, our experimental results indicate that native species have low capacities to recolonize sites dominated by invasive species. In addition, along with results of “invasiveness” experiments (e.g., Xu et al., 2004; Michelan et al., 2013), our results suggest that pre-occupation is key to predicting competition effects. The negative effects on the native species derived from the pre-occupation of the exotic species are likely to be more pronounced in ecosystems subject to anthropogenic impacts, since anthropogenic impacts are more favorable to the success of invasive plants (Daehler, 2003; Havel et al., 2005; Engelhardt, 2011). In summary, we speculate that the natural recolonization by native species in environments dominated by *U. arrecta* is unlikely and that their success can only occur if the invasive species is manipulated, reducing its occupation.

Experiments in greenhouses and controlled conditions are criticized for using small spatial and temporal scales and for not replicating the complexity found in natural environments (Gibson et al., 1999). Experiments like ours could, for example, bias the effects of shoot competition because of limited soil volume, which reduces shoot growth, and because of edge effects, which allow more access to light than would occur in the field (Kiaer et al., 2013). However, the data obtained in the field for *P. cordata* demonstrate that at least the direction and intensity of the competitive effects exerted by *U. arrecta* were similar to those found in the greenhouse. This congruence suggests that the data obtained experimentally in the greenhouse can be extrapolated to field situations, as the results obtained in the latter also indicate the importance of density-dependent effects of an invasive species on native species.

Finally, we believe that the larger effect sizes in our experiment compared to those estimated by Jauni and Ramula (2015) may indicate that *U. arrecta* possesses higher competitive effects than other invasive species. The consistent negative effect of exotic plants on the biomass of native plants, according to Jauni and Ramula (2015), may be explained by considering three mechanisms that are not mutually exclusive. The first mechanism, and probably the most important, is related to competition for light and nutrients, which reduces biomass and may cause a decrease in the reproductive success and survival of native plant species, leading to population decline (Jauni and Ramula, 2015). The other two mechanisms are related to pollinators and survival of recruits, but these mechanisms cannot be used to explain our results because our experiments considered only one plant generation. Interestingly, the results obtained by Jauni and Ramula (2015) suggest that native plants can be established when associated with exotic plants. These authors suggest that the population dynamics of native plants may not be limited by the availability of micro-sites but by competition with exotic species in later stages of the life cycle. Our results corroborate this expectation, since most of the native propagules established even in high biomass of the exotic species, but they attained extremely low growth and did not flower in this condition.

In short, our hypotheses that the effects of competition between *U. arrecta* and native macrophytes are density dependent and that *P. cordata* and *L. hexandra* respond differently to this competitive interaction were corroborated. Our results suggest that in a scenario of dominance of invasive Poaceae, recolonization by native macrophytes is unlike to occur naturally. Our results also indicate that the reduction of the diversity of native macrophytes observed in sites colonized by *U. arrecta* can be explained by the competitive effects of this invasive species. In practical terms, due to the density-dependent competitive effects, when it is not possible to eliminate the invasive species, a strategy to maintain it at low density would be required to allow recolonization by natives and to maintain local biodiversity. Also in this context, we believe that increasing the number of propagules to analyze the capacity of native species to recolonize environments densely colonized by exotic species would be an interesting avenue for further research.

## Author Contributions

TM and ST conceived the ideas and designed the experiments. TM, ST, and FB conducted the greenhouse and field experiments. TM and LB analyzed and interpreted the data. TM led the writing of the manuscript. ST and LB gave major input into the first draft. All authors contributed critically to the final version of the manuscript and approved it for publication.

## Conflict of Interest Statement

The authors declare that the research was conducted in the absence of any commercial or financial relationships that could be construed as a potential conflict of interest.
