# Supplementary material for: Competitive Effects Hinder the Recolonization of Native Species in Environments Densely Occupied by One Invasive Exotic Species
Source: Front Plant Sci. 2018 Sep 4;9:1261. doi: 10.3389/fpls.2018.01261 (PMC6131817; doi:10.3389/fpls.2018.01261)
Supplement: Supplementary file 1 [file Table_1.DOCX]

Supplementary Material

**Competitive effects hinder the recolonization of native species in environments densely occupied by invasive exotic species**

**Thaisa S. Michelan*, Sidinei M. Thomaz, Fabielle M. Bando and Luis Mauricio Bini**

*** Correspondence:** Corresponding Author: tsmichelan@ufpa.br

## Supplementary Figures

## Supplementary Figure 1 – Photos of the experiments in a greenhouse and *in situ*.


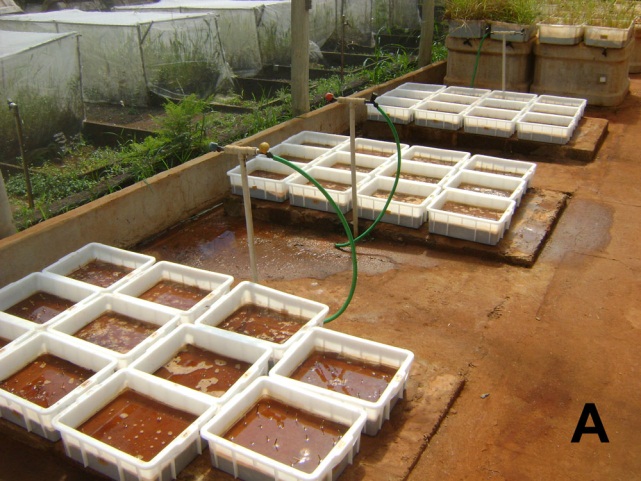

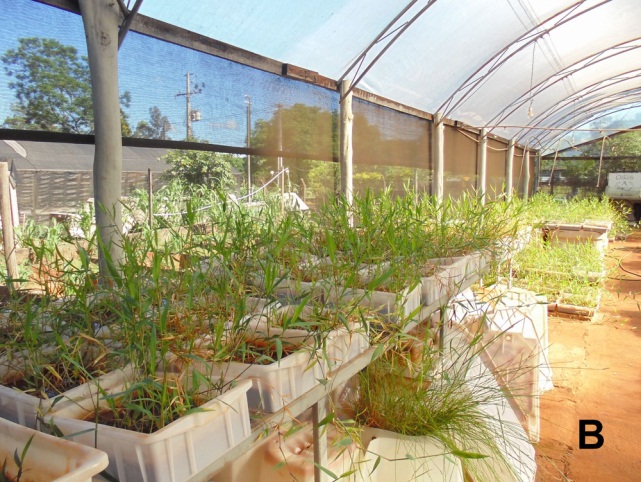

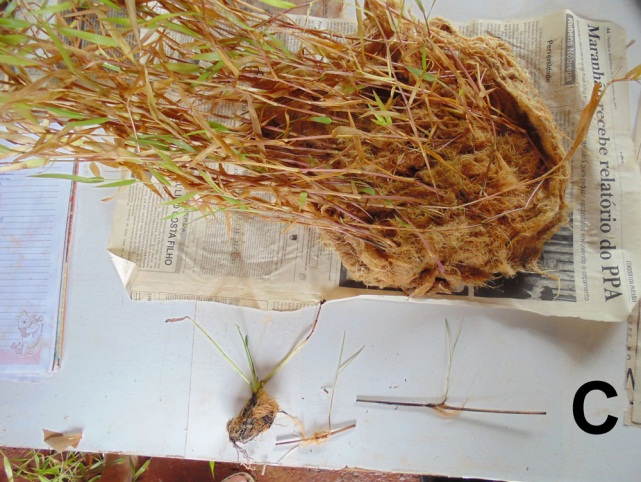

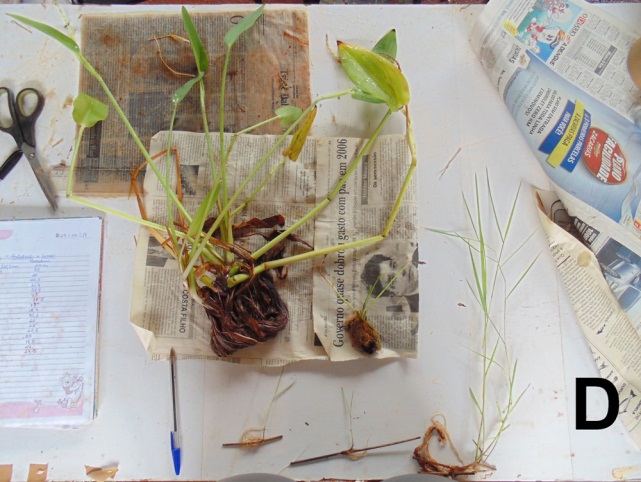

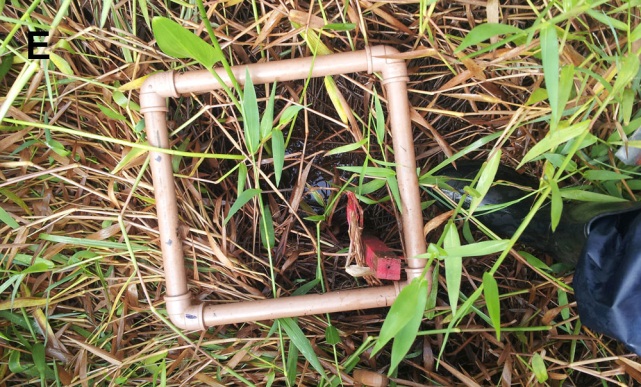

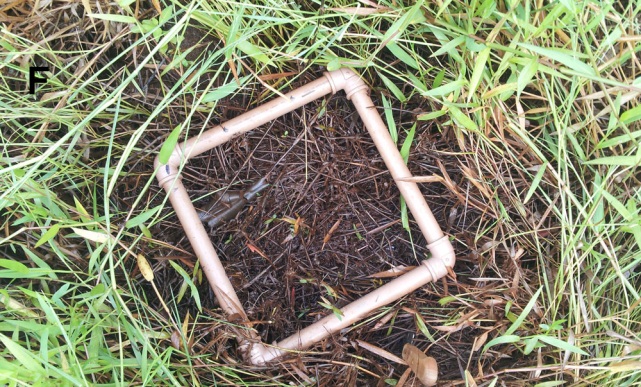


**F**

**E**

**D**

**C**

**B**

**A**

**Supplementary Figure 1.** Photos of the experiment in the greenhouse (A-D) and *in situ* (E-K). A and B - Beginning and ending of the experiment, respectively; C - in the lower part of the figure, *Pontederia cordata* and *Leersia hexandra* present in the treatment with high density of *U. arrecta*; D – Natives species that developed without (large specimens) and with (smaller specimens) competition with the invasive species; E and F - Treatment *in situ* with the native and exotic developing and after the collection of native and invasive species; To be continued.


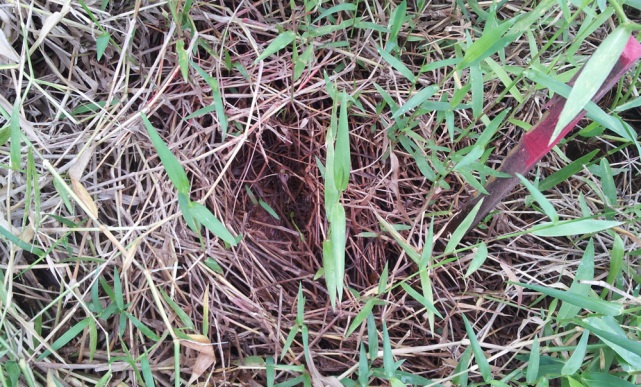

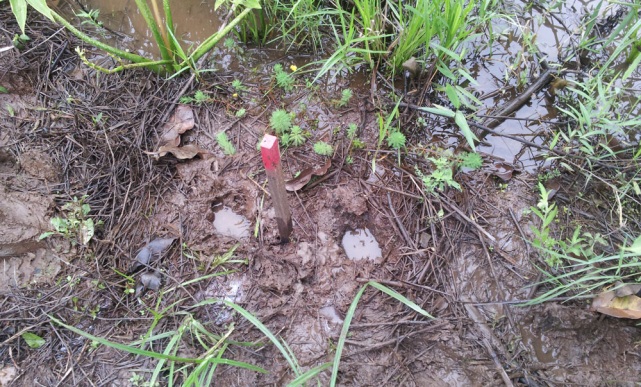


**H**

**G**


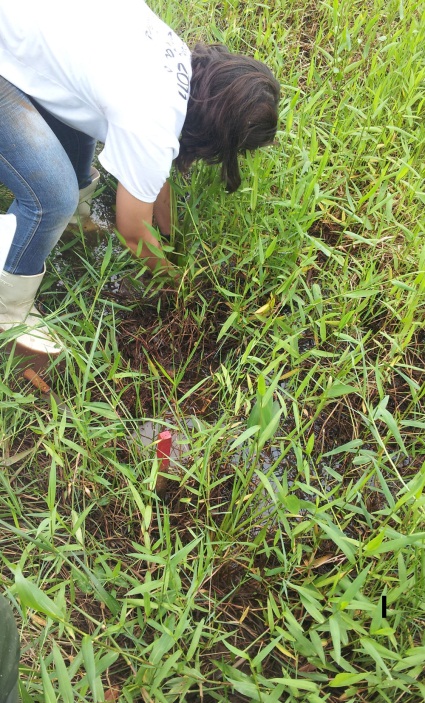

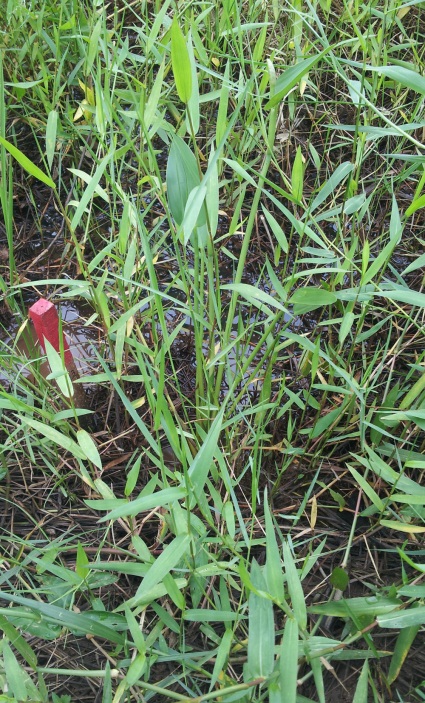

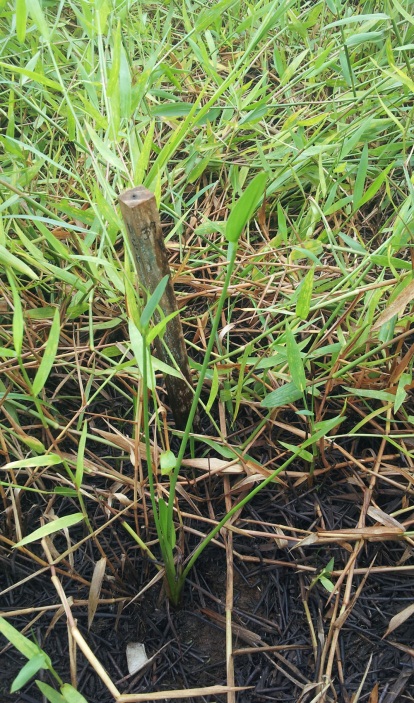


**I**

**K**

**J**

**Supplementary Figure 1.** *Continua -* G and H - Local with moderate density and absence of *U. arrecta*, respectively and I, J and K - Native and invasive species co-occurring in the experiment conducted *in situ*.

## Supplementary Figure 2 - Results based on all gradient of *U. arrecta* biomass (with addition of treatment 0 – absence of competition) from the greenhouse experiment.


**Supplementary Figure 2.** Trends in the relationships between biomass of *Urochloa arrecta* and biomass (A), length (B) and root:shoot ratios (C) of the two native species *Pontederia cordata* and *Leersia hexandra* with addition of treatment 0 in the greenhouse experiment.
